# Right-sided iliac vein compression syndrome: when the vein is compressed between the internal and external iliac arteries—a case report

**DOI:** 10.1093/ehjcr/ytae011

**Published:** 2024-01-09

**Authors:** Tetsuo Yamanaka, Tatsuhiko Ishihara, Toru Hara, Yoshimaro Ichinohe, Toru Fukatsu

**Affiliations:** Department of Cardiology, Tokyo Teishin Hospital, 2-14-23 Fujimi, Chiyoda-ku, Tokyo 102-8798, Japan; Department of Cardiology, Kanto Central Hospital of the Mutual Aid Association of Public School Teachers, 6-25-1 Kamiyouga, Setagaya-ku, Tokyo 158-8531, Japan; Department of Cardiology, Tokyo Teishin Hospital, 2-14-23 Fujimi, Chiyoda-ku, Tokyo 102-8798, Japan; Department of Cardiology, Tokyo Teishin Hospital, 2-14-23 Fujimi, Chiyoda-ku, Tokyo 102-8798, Japan; Department of Cardiology, Tokyo Teishin Hospital, 2-14-23 Fujimi, Chiyoda-ku, Tokyo 102-8798, Japan

**Keywords:** Deep vein thrombosis, Iliac vein compression syndrome, Venous thromboembolism, Right-sided iliac vein compression syndrome, Case report

## Abstract

**Background:**

In its normal anatomical relationship, the inferior vena cava is located on the right side of the abdominal aorta. Iliac vein compression syndrome (IVCS) is a pathological condition in which a blood clot is formed due to blood flow obstruction when the left common iliac vein is compressed between the right common iliac artery and the vertebral body. Therefore, right-sided IVCS (RIVCS) is rare. The effectiveness of treatment for RIVCS has not been sufficiently investigated.

**Case summary:**

A 51-year-old man developed deep vein thrombosis in the right lower extremity and non-massive pulmonary embolism during steroid treatment for IgA nephropathy. Magnetic resonance angiography (MRA) suggested iliac compression syndrome. Symptoms improved with the use of direct oral anticoagulants and compression stockings. At the 8-month follow-up, the clinical course was uneventful.

**Discussion:**

The causes of RIVCS in this case are believed to be the effects of steroids, prolonged sitting, and compression of the right external iliac vein. However, considering that deep vein thrombosis did not form in the left lower limb where there was no venous compression, it can be considered that the compression of the right external iliac vein had a significant impact. This case has been followed up for 8 months with anticoagulants and is progressing well. This is the first case to report the course of RIVCS treated conservatively with anticoagulant therapy for 8 months. This case suggested that conservative treatment is effective for RIVCS.

**Learning points**

- Iliac vein compression syndrome (IVCS) usually occurs in the left common iliac vein due to anatomical factors and is an important factor of venous thromboembolism. Iliac vein compression syndrome rarely occurs in the right iliac vein.
- Right IVCS (RIVCS) also causes venous thromboembolism, although its epidemiology and treatment have not been fully investigated. This case suggests that conservative treatment with anticoagulant therapy may be effective for RIVCS.

## Introduction

The inferior vena cava is located to the right of the abdominal aorta. Thrombi may form due to blood flow disturbances caused by compression of the left common iliac vein between the right common iliac artery and vertebral bodies. Therefore, iliac vein compression syndrome (IVCS) commonly occurs in the left lower extremity^1,2^; right-sided IVCS (RIVCS) is rare.^3–7^ Furthermore, treatment methods and prognoses have been insufficiently studied.

## Summary figure

**Figure:**
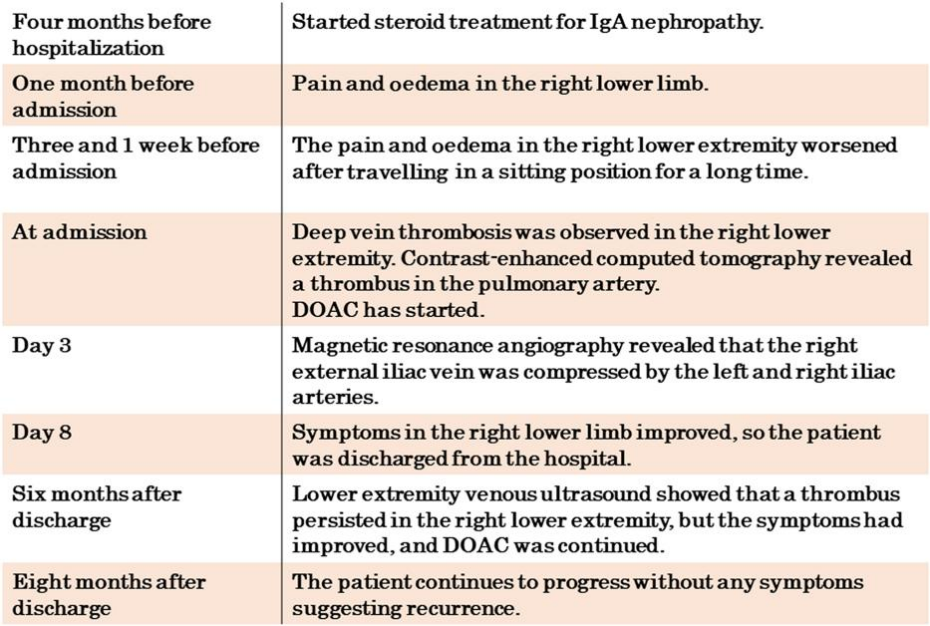


## Case presentation

We report the case of a 51-year-old man diagnosed with chronic kidney disease (CKD) caused by IgA nephropathy at 50 years. Steroid treatment for IgA nephropathy was initiated 4 months before admission. One month before admission, he noticed pain and oedema in his right lower limb. The patient visited our hospital because of worsening pain and oedema after sitting for long periods of time, both 1 and 3 weeks before presentation. There was no chest pain or shortness of breath during exertion. Blood pressure, pulse, and percutaneous arterial oxygen saturation at room air were 150/86 mmHg, 71 b.p.m., and 98%, respectively. Auscultation revealed no obvious abnormalities in heart or respiratory sounds. No jugular vein distention was observed. The patient reported oedema from the right thigh to the lower leg and grasping pain on the dorsum of the lower leg. The patient had a score of 8 points on the Villalta scale, with 2 points each for pain and heaviness, anterior tibial oedema, and grasping pain of the lower leg.

The patient’s platelet count, d-dimer, creatinine, albumin, and high-sensitivity troponin levels were 20.8 × 104/µL (reference: 14.6–34.8 × 104/µL), 17.2 µg/mL (reference: 0.00–1.00 µg/mL), 1.79 mg/dL (reference: 0.65–1.07 mg/dL), 3.8 g/dL (reference: 3.9–4.9 mg/dL), and 0.02 ng/mL (reference: 0.00–0.04 ng/mL), respectively. Twelve-channel electrocardiography showed a sinus rhythm with a pulse of 57 b.p.m. and was otherwise unremarkable. Chest radiography revealed a cardiothoracic ratio of 41% but no cardiomegaly or abnormalities in the lung fields. Transthoracic echocardiography revealed a left ventricular ejection fraction of 62%, a trans-tricuspid pressure gradient of 19 mmHg, and no right atrial or ventricular enlargement.

**Figure 1:**
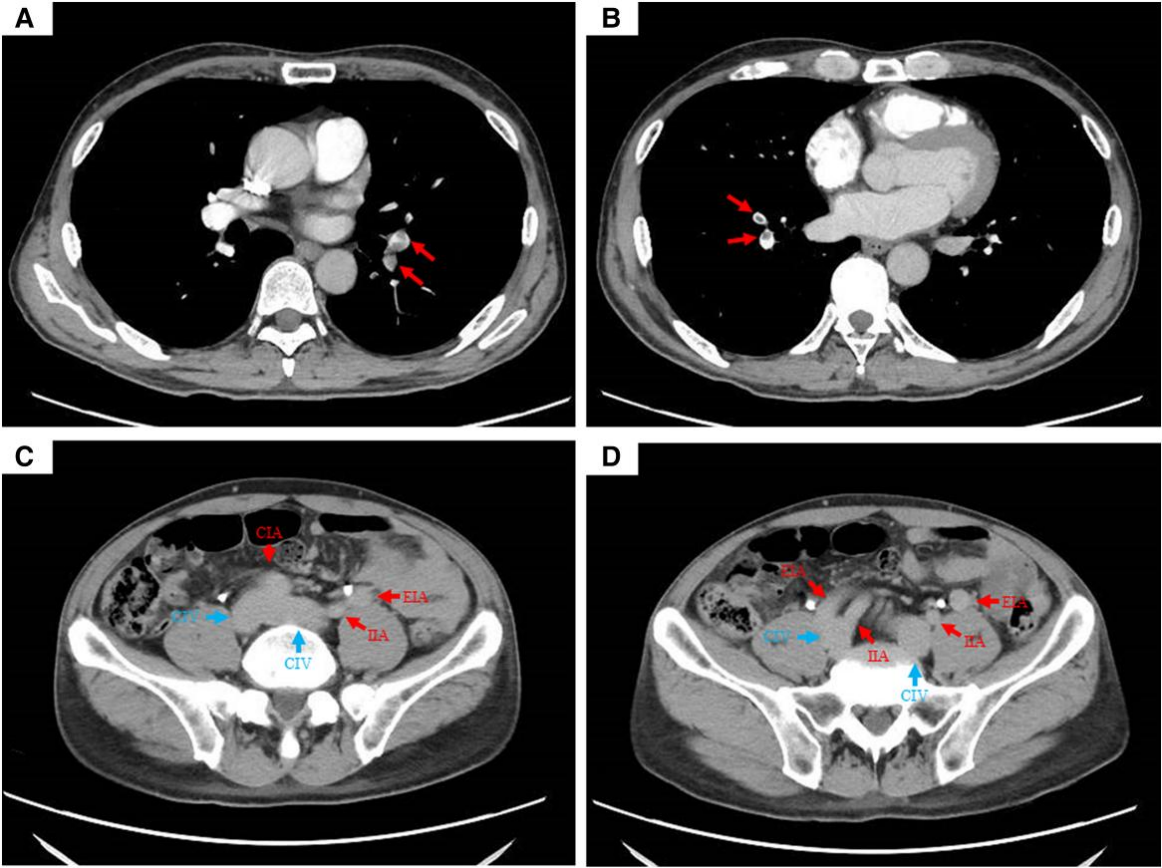
Contrast-enhanced computed tomography. (*A*, *B*) Multiple thrombi in both pulmonary arteries (arrow). (*C*, *D*) No compression of the left common iliac vein was observed. The right external iliac vein ran between the right internal iliac artery and external iliac artery but could not be adequately evaluated. CIA, common iliac artery; CIV, common iliac vein; EIV, external iliac vein; IIA, internal iliac artery; EIA, external iliac artery.

The patient had CKD; therefore, to prevent contrast agent nephropathy, 0.9% normal saline was administered at 1 mL/kg/h from 2 h before to 24 h after contrast agent administration. Subsequently, contrast-enhanced computed tomography (CT) was performed, revealing multiple thrombi in both pulmonary arteries (*Figure 1A* and *B*).

**Figure 2:**
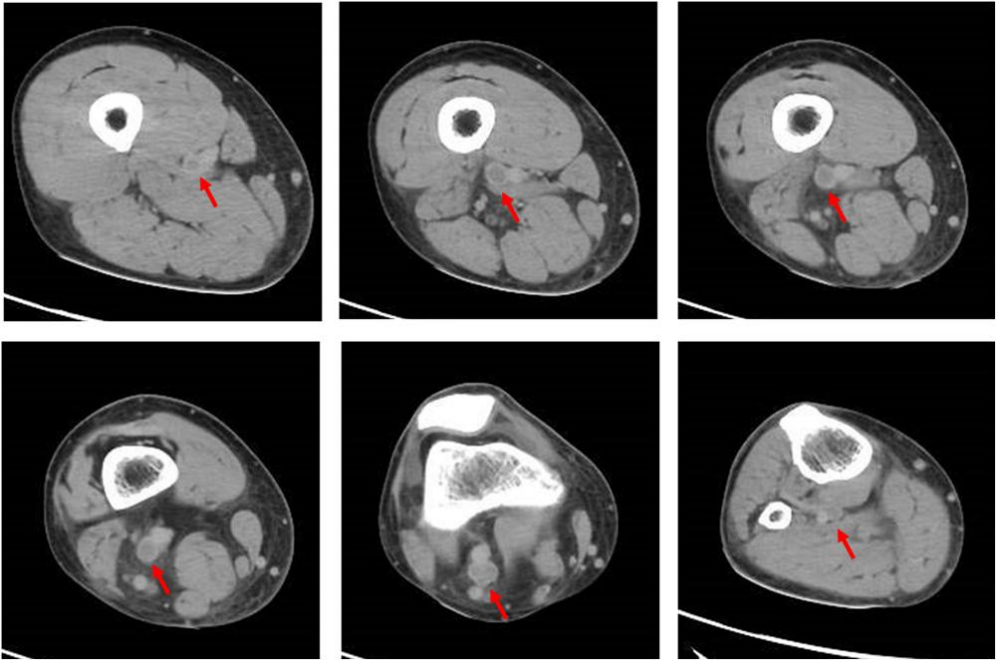
Venous phase of contrast-enhanced computed tomography. Thrombus was observed from the distal side of the right femoral vein to the deep leg vein (Red arrows).

No compression of the left common iliac vein was observed (*Figure 1C*). The right external iliac vein (EIV) ran between the right internal iliac artery (IIA) and external iliac artery (EIA), but its evaluation was insufficient (*Figure 1D*). Imaging obtained during the venous phase revealed a thrombus from the distal side of the right femoral vein to the deep leg vein (*Figure 2*). No neoplastic lesions were observed from the chest to the pelvis.

The patient was diagnosed with deep vein thrombosis (DVT) and non-massive pulmonary embolism (PE) equivalent to a simplified PE severity index of 0 and was admitted on the same day for further investigation and treatment.

The antithrombin, plasminogen, protein C, protein S, fibrinogen, tissue plasminogen activator/plasminogen activator inhibitor-1 complex, total homocysteine, anticardiolipin-β2 glycoprotein-I complex antibody, and antinuclear antibody levels were within the normal range.

**Figure 3:**
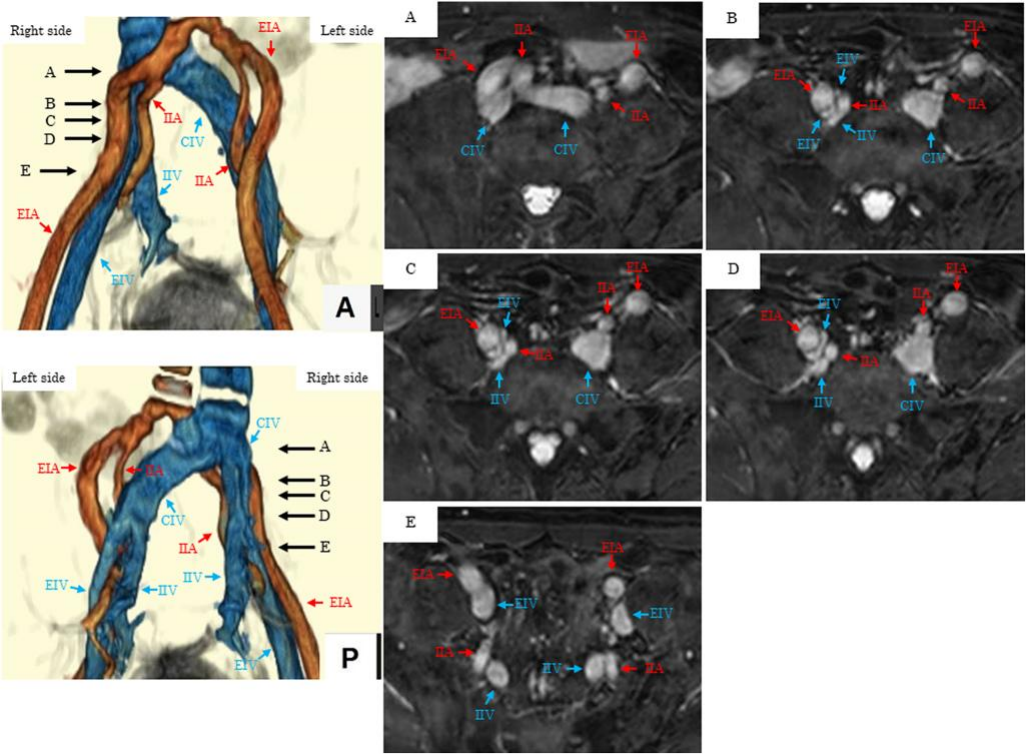
Lower extremity MRA. The right external iliac vein was compressed by the right internal iliac artery and external iliac artery. Quantitative evaluation showed that the distal external iliac vein maximum minor diameter was 13.3 mm (*E*) and that the maximum compression diameter was 2.1 mm (*B*), indicating severe stenosis (84%). %stenosis = (maximum minor diameter − maximum compression diameter)/maximum minor diameter). EIV, external iliac vein; IIA, internal iliac artery; EIA, external iliac artery.

After hospitalization, anticoagulant therapy with direct oral anticoagulants (DOAC; edoxaban 30 mg) was initiated, and compression stockings were applied. Lower extremity magnetic resonance angiography (MRA) without contrast agents was performed on Day 3. This revealed compression of the right EIV by the right IIA and EIA (*Figure 3*). On quantitative evaluation, the distal EIV maximum minor diameter was 13.3 mm and maximum compression diameter was 2.1 mm, indicating severe stenosis (84%).

Following treatment, the lower extremity pain and oedema resolved. Upper gastrointestinal endoscopy and human haemoglobin tests were performed on Day 8, with no abnormal findings.

**Figure 4:**
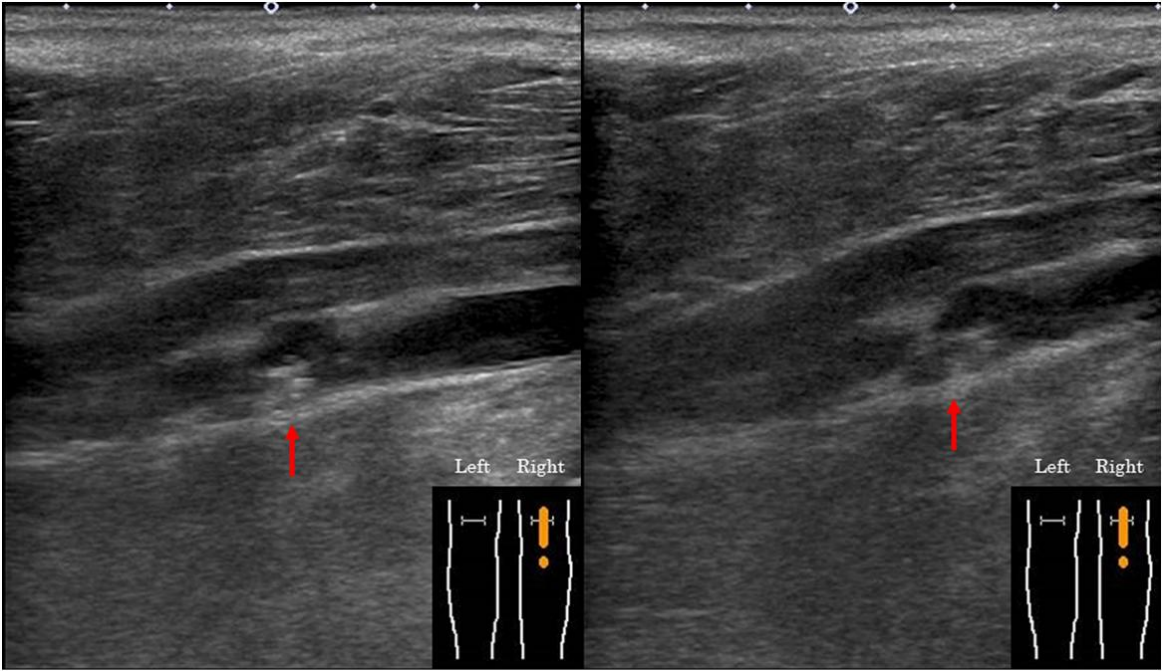
Lower extremity ultrasonography 6 months after discharge. Residual thrombus was observed (arrow).

By that point, the patient’s d-dimer level improved to 2.2 µg/mL, and his symptoms had significantly improved (Villalta scale 1, with 1 point for anterior tibial oedema). Furthermore, he declined to undergo treatment with a stent and was discharged from the hospital, with a plan to consider invasive treatments, such as a stent placement, if his condition showed any signs of deterioration.

d-Dimer levels normalized to 0.1 g/mL at 1.5 months after discharge and remained at <0.1 µg/mL thereafter. Nonetheless, despite the absence of dyspnoea or lower limb oedema, lower extremity ultrasonography performed 6 months later revealed residual thrombus (*Figure 4*).

Once again, we proposed catheterization with a stent, but the patient declined this option. Consequently, we continued therapy with edoxaban 30 mg. Thereafter, conservative treatment with edoxaban 30 mg was performed until 8 months after discharge from hospital, but the patient’s clinical course was uneventful, with no recurrence of lower limb oedema, pain, or dyspnoea. Furthermore, since the patient’s ongoing treatment for IgA nephropathy involved steroids and RIVCS persisted, we plan to continue the DOAC therapy.

## Discussion

Neither congenital thrombotic predisposition nor antiphospholipid syndrome was identified as the cause of DVT in this case. There was no history of smoking, and no neoplastic lesions were detected. Therefore, we concluded that the DVT occurred due to a combination of three acquired factors. First is the effect of steroid-induced hypercoagulability in IgA nephropathy. There is a three-fold increased risk of DVT in patients undergoing steroid treatment.^8^ In this case, steroid therapy was initiated 4 months before symptom onset and was suggestive of DVT. Second is the blood flow stagnation resulting from prolonged sitting. Three weeks and 1 week before the current diagnosis, he had travelled long distances for several hours in a sitting position, after which his symptoms exacerbated. Third is the impact of IVCS. The right EIV was compressed between the right EIA and IIA on MRA. Iliac vein compression syndrome reportedly occurs in 62% of DVT cases and is considered an important pathological condition causing DVT.^1^

Usually, the IVC is to the right of the abdominal aorta. A thrombus is formed due to blood flow disturbance caused by the left common iliac vein being compressed between the right common iliac artery and vertebral body. Therefore, IVCS commonly occurs in the left lower extremity,^1,2^ and RIVCS has rarely been reported. We identified only two multiple case studies^3,4^ and three case reports^5–7^ on RIVCS.

Chen *et al.*^3^ reviewed 16 cases of RIVCS and categorized them into three types (*Table 1*). In addition, Chen *et al.*^4^ investigated iliac compression in 32 cases of RIVCS and classified them into three types (*Table 2*). Considering these reports,^3,4^ this case was considered equivalent to type II or type (a). In 37.5^3^ or 43.75%^4^ of the patients, the RIV was compressed between the IIA and EIA, as in the present case. Chen *et al.*^4^ also observed that the stenosis rate was significantly higher for the right-sided DVT (48.54%) than for the left-sided DVT (22.29%). A mechanism similar to that in this case has been reported.^5,6^ Furthermore, a case of right common iliac vein compression by the right IIA has been reported,^7^ and various mechanisms may be considered besides the classifications.^3,4^

**Table 1:** The 3 types of right-sided iliac vein compression syndrome

Type I	Compression by the right common iliac artery and lumbar spine.
Type II	Compression at the intersection angle between the bifurcation of the right external and internal iliac arteries.
Type III	Compression by the right EIA alone.

**Table 2:** Three anatomical right iliac vein compression patterns

Type (a)	Right iliac vein (RIV) sandwiched between the right EIA.
Type (b)	Right IIA, RIV compressed by the right EIA against the sacrum.
Type (c)	RIV compressed by the right common iliac artery and right IIA against the psoas major muscle.

The incidence of DVT is 3.03-fold higher in cases with ≥70% stenosis.^9^ Here, there was 84% stenosis. In this case, DVT did not develop in the left lower extremity without iliac vein compression under the same systemic conditions of steroid use and long-term sitting. Deep vein thrombosis was observed only in the right lower extremity, with 84% stenosis of the EIV between the right IIA and EIA. Therefore, we considered that RIVCS may have contributed to DVT development.

In principle, anticoagulant therapy is recommended for PE/DVT treatment in patients with low to intermediate risk of PE/DVT with normal blood pressure and no right ventricular dysfunction.^10^ Therefore, anticoagulant therapy with DOAC was commenced.

In cases where the risk factor is reversible, discontinuation of anticoagulant therapy for 3 months is recommended. However, in cases where the risk factors persist, anticoagulant therapy should be continued indefinitely.^10^

Regarding the treatment of ‘compressed’ RIVCS, such as this case, Chen *et al.*^3^ reported that 15 patients included in their study were treated with a stent and that patency was confirmed by CT in all patients with an average follow-up of 28.5 ± 3.6 months. Additionally, treatment with a stent can be effective in the acute phase of DVT.^6^ Toguchi *et al.*^5^ reported persistence of the thrombus with conservative treatment with warfarin without stent usage, although the clinical outcomes were not exacerbated. However, the progress through long-term follow-up has not been reported.

Based on findings from previous reports,^3,6^ stent treatment is considered useful for managing RIVCS, and we also considered this option. However, our patient had CKD equivalent to stage 3b, making the use of iodinated contrast agents challenging. Further, the patient declined invasive treatments such as stent placement. The patient’s PE was non-massive with a PE severity index of 0, and his clinical course was uneventful. Therefore, a conservative approach involving DOAC therapy was used. However, based on this progress, we considered invasive treatment. Given that this patient required continued steroid treatment and the possibility of RIVCS being persistent, the risk of venous thromboembolism will also remain. Therefore, continued DOAC therapy is necessary in accordance with the guidelines.^10^

## Conclusion

The effectiveness of conservative treatment for RIVCS has not been sufficiently investigated. Furthermore, follow-up observation after the acute stage has not been reported. To the best of our knowledge, this is the first case to report the course of RIVCS treated conservatively with anticoagulant therapy for 8 months. This case progressed well with conservative treatment using anticoagulant therapy, suggesting that conservative treatment is effective for RIVCS.

## Data Availability

The data underlying this article will be shared upon reasonable request to the corresponding author.

## Lead author biography

Dr Tetsuo Yamanaka started his career in Tokyo Teishin Hospital, Tokyo, Japan. After working in the Department of Cardiovascular Medicine at Sakakibara Heart Institute, he has been a cardiologist in the Department of Cardiovascular Medicine at Tokyo Teishin Hospital, Tokyo, Japan, since 2014, where he provides treatment for a wide range of cardiovascular diseases, including endovascular treatment.
